# Human Lower Limb Joint Biomechanics in Daily Life Activities: A Literature Based Requirement Analysis for Anthropomorphic Robot Design

**DOI:** 10.3389/frobt.2020.00013

**Published:** 2020-02-11

**Authors:** Martin Grimmer, Ahmed A. Elshamanhory, Philipp Beckerle

**Affiliations:** ^1^Lauflabor Locomotion Laboratory, Department of Human Sciences, Institute of Sports Science, Technische Universität Darmstadt, Darmstadt, Germany; ^2^Mechanical Engineering, Technische Universität Darmstadt, Darmstadt, Germany; ^3^Elastic Lightweight Robotics Group, Department of Electrical Engineering and Information Technology, Robotics Research Institute, Technische Universität Dortmund, Dortmund, Germany; ^4^Institute for Mechatronic Systems, Mechanical Engineering, Technische Universität Darmstadt, Darmstadt, Germany

**Keywords:** movement, biomechanics, lower limb, daily activity, human, joint, power, moment

## Abstract

Daily human activity is characterized by a broad variety of movement tasks. This work summarizes the sagittal hip, knee, and ankle joint biomechanics for a broad range of daily movements, based on previously published literature, to identify requirements for robotic design. Maximum joint power, moment, angular velocity, and angular acceleration, as well as the movement-related range of motion and the mean absolute power were extracted, compared, and analyzed for essential and sportive movement tasks. We found that the full human range of motion is required to mimic human like performance and versatility. In general, sportive movements were found to exhibit the highest joint requirements in angular velocity, angular acceleration, moment, power, and mean absolute power. However, at the hip, essential movements, such as recovery, had comparable or even higher requirements. Further, we found that the moment and power demands were generally higher in stance, while the angular velocity and angular acceleration were mostly higher or equal in swing compared to stance for locomotion tasks. The extracted requirements provide a novel comprehensive overview that can help with the dimensioning of actuators enabling tailored assistance or rehabilitation for wearable lower limb robots, and to achieve essential, sportive or augmented performances that exceed natural human capabilities with humanoid robots.

## 1. Introduction

A variety of robots are already a part of most industrial production lines, e.g., robotic arms are used to move and manipulate objects. Humans like to increase robot capabilities to assess the potential for robotic assistance in daily life. For daily life assistance, service robots (Mende et al., 2019) or social robots (Góngora Alonso et al., 2019) would need to move in urban environments and they require to use objects made for humans (e.g., cars, kitchen equipment). Many of these objects are adapted to the human body composition. Thus, it could be advantageous to design robots with an anthropomorphic structure, including human-like lower limbs. In addition to designing such humanoid robots (Kuindersma et al., 2016; Yang et al., 2017; Spenko et al., 2018), wearable lower limb robots, including exoskeletons for rehabilitation (Miller et al., 2016; Moreno et al., 2018; Chen et al., 2019; del Carmen Sanchez-Villamañan et al., 2019) and daily assistance (Schmidt et al., 2017; del Carmen Sanchez-Villamañan et al., 2019; Grimmer et al., 2019a; Kapsalyamov et al., 2019) or prostheses (Au et al., 2009; Versluys et al., 2009; Cherelle et al., 2016; Grimmer et al., 2016; Pieringer et al., 2017), are of interest. Instead of moving independently like humanoid robots, lower limb wearable robots for those with walking capability have to move in conjunction with the human lower limbs. Thus, the movement capabilities of the wearable robots have to at least match the human movement capabilities. To achieve such a matching, human anthropometric information (e.g., segment lengths, masses, inertia values) and the joint capabilities must be identified to deduce the robotic system specifications. While summaries of anthropometric data can already be found in literature (Winter, 2009), it is rare to find a comprehensive overview of performance values for human lower limb joints.

For the identification of joint performance dynamometers can be used, which can determine the relationship between maximum joint moment and maximum joint speed (Drouin et al., 2004). These analyses could be performed with different populations in terms of age (e.g., students or elderly people) or with populations that have different levels of physical athleticism (e.g., athletes or non-athletes). However, knowledge of maximum joint performance values would not provide us with insights regarding joint requirements throughout daily life. Designing robots based on the human performance maxima, might overestimate the required specifications, which could lead to disadvantages such as increased weight and reduced operating time. An alternative would be to analyze lower limb joint performance during human daily movements.

Humans have developed highly versatile movement skills (Torricelli et al., 2015). This includes a wide range from minimal movements as in maintaining balance during quite stance over movements in place when for example lifting objects, to movements that are used to ambulate such as walking. All movement tasks can be varied in several dimensions, which will change their biomechanical characteristic. For example, one task could be to design a powered prosthetic ankle that is able to assist a person with a transtibial amputation during walking. Walking is determined by the velocity, the slope of the environment, and the shape of the ground below the foot. Moreover, during locomotion and movements in place, differences in body weight and additional payload could be considered, as both will change the human joint effort. A similar scaling effect might exist for movements that are performed by two legs, compared to movements that completely rely on a single leg.

With this study we intend to summarize and analyze the hip, knee, and ankle joint kinematics and kinetics for a broad range of daily essential and sportive movements. Instead of performing several biomechanical movement experiments on our own, available data from literature is used for the analysis. We aim to identify the most demanding movements in terms of maximum absolute joint angular velocity, maximum absolute joint angular acceleration, maximum absolute joint moment, maximum absolute joint power, average absolute power, and joint range of motion. Additionally, this study is used to investigate if there exist differences in the maxima of locomotion tasks for the stance and the swing phase, as this could allow alternative mechanical solutions to mimic either of these phases. We expect that the non-weight bearing swing phase has increased angular velocity and angular acceleration requirements, whereas the weight bearing stance phase has increased requirements in moment and power. The identified maxima will be discussed and compared to human performance limits. An outlook will be provided on how to reduce the identified requirements with a variety of mechanical solutions. The extracted hip, knee, and ankle joint kinematics and kinetics will be provided for future use as Appendix.

## 2. Materials and Methods

### 2.1. Selection of the Biomechanical Studies

Articles with biomechanical data were selected based on the following requirements:

1. Studies had to report biomechanical data of the hip, knee, and ankle joint.
2. The published data of the joints had at least to include the joint angle, the joint moment, and a movement time information.
3. Biomechanical data was averaged for a larger group of individuals.

If available, joint power, joint angular velocity, and joint angular acceleration were also extracted from the articles. If the latter information was not available, we calculated it based on the published data. Moreover, the investigated populations should be younger adults without mobility related impairments and athlete data was avoided if possible.

### 2.2. Selection of the Movement Tasks

As humans perform a variety of movement tasks throughout daily life, we acknowledge that we cannot include all possible lower limb movement tasks in our analysis. However, we aimed to analyze movement tasks that are essential and common to daily life (level walking, climbing, stair ambulation, cycling, recovery, lifting, sit to stand) as well as sportive movement tasks (running, squat jump) as explained subsequently.

#### 2.2.1. Level Walking

Walking speeds (1.1 m/s, 1.6 m/s, Lipfert, 2010; Grimmer and Seyfarth, 2014) around preferred walking speed (Grimmer et al., 2019b) were selected as a representative speed for typical daily walking speeds.

#### 2.2.2. Climbing

In addition to level locomotion, ascending, and descending (walking) of 21°, slopes are included (Lay et al., 2006), as increased moment and power requirements and changes in range of motion are expected compared to level walking.

#### 2.2.3. Stair Ambulation

It was estimated that humans climb about 47–66 stairs a day (Grimmer and Seyfarth, 2014). While this is only a small proportion of the daily strides (7,000–13,000, Tudor-Locke and Myers, 2001), stair ambulation represents a movement task that must be considered to locomote in human made environments due to increased requirements. For our analysis, stair ascending and stair descending at a regular stair slope of 30° were included (Riener et al., 2002).

#### 2.2.4. Running

Two running speeds, 2.6 m/s and 4.0 m/s, were included (Lipfert, 2010; Grimmer and Seyfarth, 2014). The lower running speed is also referred to as jogging, based on the arbitrary definition of (Larsen et al., 2002). To put these running speeds in context, about 10% of younger marathon runners (Zavorsky et al., 2017) ran at a speed below 2.6 m/s (finishing time 272 min) and about 10% ran at a speed above 4.0 m/s (finishing time 176 min). Thus, the considered data covers the majority of recreational runners.

#### 2.2.5. Cycling

A popular human means of transport is the bicycle. Up to 7.4% of survey participants from a US study (Nelson and Allen, 1997) used bicycles for commuting. Therefore, we included cycling at an ergometer at 120 W (Ericson et al., 1986) in the analysis.

#### 2.2.6. Recovery

As walking is our primary locomotion task, and 73% of falls occur during walking (Do et al., 2015), we aimed to also include a representative movement task that is used to avoid falling. Fifty-seven percent of falls are caused by slipping, tripping, or stumbling (Schiller et al., 2007). To avoid falling after stumbling, a recovery movement is required to stabilize the body. We therefore included a recovery movement that was provoked under lab conditions (Hsiao-Wecksler and Robinovitch, 2007).

#### 2.2.7. Squat Jump

While being a rare activity for elderly people, we decided to include jumping in order to have a representative task that has an explosive quality and that begins in stance. Jumping can be used to reach elevated objects or to pass obstacles. It is also a fundamental activity in athletics (long- and high jump) as well as in ballgames such as volleyball or basketball. Therefore, a standardized squat jump movement was included in our analysis (Mackala et al., 2013). Due to missing biomechanical data for a non-athlete population, a study with a athlete population was selected for the jumping analysis (squat jump). Subjects were sprinters with a 100 m performance of 10.87 s.

#### 2.2.8. Lifting

In addition to gait-related movements, we aimed to include bending and lifting movements, as industrial workers were found to perform between 0 and 58 min of daily forward bending time (Lagersted-Olsen et al., 2016). Squat and stoop lifting with a weight of 15 kg was included (Hwang et al., 2009).

#### 2.2.9. Sit to Stand

Due to the second point of support at the beginning (e.g., chair), sit to stand transitions might exhibit increased requirements, compared to lifting an external mass, to move the center of mass above the center of pressure. Therefore, we included the sit to stand transition (Roebroeck et al., 1994) in our analysis.

### 2.3. Movement Data

The experimental equipment, selected analysis methods, and subject characteristics of the involved studies are shown in Tables 1, 2. All studies used force sensors to determine 3D ground reaction forces, e.g., an instrumented treadmill or staircase, force plates on the ground or a force sensor in the pedal of the bicycle. Most studies determined kinematic data with 3D motion capture systems. In sit to stand transition and cycling, a 2D analysis with a video camera was performed. For those studies that reported the kinetic measurement frequencies, frequencies ranged from 100 to 1,200 Hz. Reported kinematic measurement frequencies were in between 60 and 240 Hz for the 3D and in between 40 and 60 Hz for the 2D measurements. For the different movement tasks, a variety of filter methods was reported for the kinematics and kinetics. For those who reported, joint angles were either calculated in the sagittal plane or in the limb plane determined by the foot rotation in the transversal plane. All studies used inverse dynamics to determine the joint moments. While 6 to 26 subjects were involved to determine the overall mean values, subject means were determined based on 1 to 166 movement repetitions.

**Table 1:** Methods and experimental setup to acquire the biomechanics for walking (Lipfert, 2010; Grimmer and Seyfarth, 2014), climbing (Lay et al., 2006), stair ambulation (Riener et al., 2002), jogging and running (Lipfert, 2010; Grimmer and Seyfarth, 2014), cycling (Ericson et al., 1986), recovery (Hsiao-Wecksler and Robinovitch, 2007), squat jump (Mackala et al., 2013), lifting (Hwang et al., 2009), and the sit to stand transition (Roebroeck et al., 1994).

**Movement**	**Kinematics**	**Kinetics**	**Inverse dynamics**	**Rep**.
Walking	8 3D MoCap, Qualisys, 240 Hz; zl 2nd order BW 40 Hz, additionally zl 2nd order BW 10 Hz for ang. vel. and ang. acc.; angles SP	3D instrumented treadmill, ADAL-WR, HEF Techmachine, 1,000 Hz; after ID moments filtered low-pass 15 Hz	based on Günther et al. (2003), include constant segment lengths and wobbling masses	21–72
Climbing (asc., desc.)	6 3D MoCap, Peak Performance Technologies, 60 Hz; quintic spline interpolation technique (Woltring, 1986); angles LP	custom walkway 3.11 m, 3D forceplate, 1,200 Hz, 4th order zl BW 25 Hz	methods from Winter (1980); Vaughan et al. (1999)	5
Stairs (asc., desc.)	4 video camera ELITE system (Ferrigno and Pedotti, 1985), 100 Hz; low-pass filtered by a model-based bandwidth-selection procedure (D'amico and Ferrigno, 1990); angles LP	4 step 3D instrumented staircase, Bertec, 100 Hz; filtering nr	short description of inverse dynamics	5
Jogging, Running	similar to walking	similar to walking	similar to walking	up to 166
Cycling	1 perpendicular video camera, Paillard Bolex, 60 Hz, analyzed at 24 Hz; switch to determine revolution; filtering nr; angles in pedal plane (SP)	3D instrumented left pedal of bicycle ergometer, Cardionic, frequency nr, time resolution 1 ms; filtering nr	based on Bratt and Ericson (1985)	1
Recovery	6 3D MoCap, MacReflex, Qualisy, 60 Hz; recursive, 4th order BW 6 Hz; angles SP	3D force plate in walkway, 6090H, Bertec, 960 Hz; 4th order BW 96 Hz	based on Cappozzo et al. (1975)	4
Squat Jump	6 3D MoCap, TVC BTS Smart-E, BTS Bioengineering, 120 Hz; filtering nr; angles SP	3D force plate for each limb, 9286B, Kistler, 240 Hz; filtering nr	nr	3
Lifting (squat, stoop)	3D MoCap, Vicon, frequency nr; filtering nr; angles nr	3D force plate for each limb, Kistler, frequency nr; filtering nr	based on Winter (1980) and Hof (2000)	nr
Sit to Stand	1 perpendicular video camera, Teledyne DBM 55, Arcadia, 40 Hz; 2nd order zl BW 4 Hz; angles SP	3D forceplate under right foot, 9281B, Kistler, 250 Hz; low-pass filter 4th order 75 Hz	based on Winter (1979)	5

*Rep. defines the number of used repetitions or strides per subject to determine the subject mean. BW is Butterworth filter, zl is zero-lag, MoCap is motion capture cameras. LP indicates that joint angles were determined in the limb plane determined by the foot rotation in the transversal plane. SP indicates that joint angles were determined in the sagittal plane. Unreported information is indicated by nr*.

**Table 2:** Subject characteristics for walking (Lipfert, 2010; Grimmer and Seyfarth, 2014), climbing (Lay et al., 2006), stair ambulation (Riener et al., 2002), jogging and running (Lipfert, 2010; Grimmer and Seyfarth, 2014), cycling (Ericson et al., 1986), recovery (Hsiao-Wecksler and Robinovitch, 2007), squat jump (Mackala et al., 2013), lifting (Hwang et al., 2009), and the sit to stand transition (Roebroeck et al., 1994).

**Movement**	**Number of**	**Age**	**Height**	**Mass**	**Gender**
	**subjects [n]**	**[yrs]**	**[m]**	**[kg]**	**male, female**
Walking	21	25.4 ± 2.7	1.73 ± 0.09	70.9 ± 11.7	10, 11
Climbing (asc., desc.)	9	24 ± 3	1.78 ± 0.08	73.36 ± 8.6	5, 4
Stairs (asc., desc.)	10	28.8 ± 2.9	1.79 ± 0.05	82.2 ± 8.5	10, –
Jogging	21	25.4 ± 2.7	1.73 ± 0.09	70.9 ± 11.7	10, 11
Running	7	23.7 ± 1.1	1.8 ± 0.1	77.5 ± 8.8	6,1
Cycling	6	25.3	1.8 ± 0.06	71.3 ± 5.0	6, –
Recovery	10	28 ± 4	1.63 ± 0.07	62 ± 10	–, 10
Squat Jump	6	21.6 ± 2.7	1.86 ± 0.05	78.16 ± 8.15	6, –
Lifting (squat, stoop)	26	23.5 ± 0.76	1.72 ± 0.06	66.5 ± 6.4	26, –
Sit to Stand	10	27.0 ± 3.5	1.76 ± 0.1	67.8 ± 10.5	4, 6

#### 2.3.1. Data Preparation

As presented in Table 3, most of the data was digitized with the software ScanIt (amsterCHEM, Almería, Spain) from the selected literature. ScanIt is a software that allows a user to extract data points from published graphs. After the manual selection of points throughout each graph, the software equally distributes and interpolates the data points over the whole time series, resulting in 200 data points for each graph from which data were extracted. Matlab was then used to filter the data with a zero-lag, 4th order Butterworth filter with a cutoff frequency between 2 and 7 Hz for the angle data, 4 and 5 Hz for the angular velocity data, 3 and 14 Hz for the moment data, and 4 to 13 Hz for the power data. To maintain the ankle moment of the impact at touch down, the recovery ankle moment was filtered at 30 Hz. The cutoff frequency was selected manually by visual inspection of each curve with the primary aim of preserving the amplitudes of the values, though also with the intention of smoothing the manually digitized data as well as possible. No additional filtering was applied before or after calculating the angular velocities and accelerations.

**Table 3:** Availability of biomechanical data for the selected movement tasks.

**Movement**	**Angle**	**Angular**	**Angular**	**Moment**	**Power**	**Time**
		**velocity**	**acceleration**			
Walking	orig	orig	orig	orig	orig	orig
Climbing (asc., desc.)	dig	calc	calc	dig	calc	dig
Stairs (asc., desc.)	dig	calc	calc	dig	dig	dig
Jogging	orig	orig	orig	orig	orig	orig
Running	orig	orig	orig	orig	orig	orig
Cycling	dig	dig	calc	dig	dig	dig
Recovery	calc	dig	calc	dig	dig	dig
Squat Jump	dig	calc	calc	dig	dig	dig, calc
Lifting (squat, stoop)	dig	calc	calc	dig	dig	calc
Sit to Stand	dig	dig, calc	calc	dig	calc	dig

*Selected studies should contain hip, knee, and ankle joint angle and joint moments and the movement time to be able to digitize (dig) them. To our advantage, joint power was mostly provided in the selected studies and had to be calculated for some movements. Walking, jogging and running data was available as an original (org) source from one of the author's lab. In most cases, angular velocity and angular acceleration had to be calculated (calc) based on the available data. Due to low frequency noise in the digitized sit to stand joint angular velocity, we decided to not use the digitized angular velocity data. Instead, we calculated joint angular velocity and acceleration based on the digitized angle data*.

The only data without angle information (Table 3) was the recovery movement (Hsiao-Wecksler and Robinovitch, 2007), in which case joint angles were calculated by numerical integration of the given angular velocities. Joint angular velocity and joint angular acceleration were calculated with numerical differentiation of the angle and angular velocity data, respectively. The duration of each activity that was used for either integration or differentiation is shown in Table 4. Joint power was calculated by multiplying the angular velocity and the joint moment for each joint. If not already normalized to body mass in the digitized graphs, joint moment and power were normalized to body mass. For recovery, joint kinetics were denormalized by the mean body height data provided in the study in order to exclude the effects of body height (Hsiao-Wecksler and Robinovitch, 2007).

**Table 4:** Time characteristics of the analyzed movements.

**Movement**	**Total time [s]**	**Time stance [%]**	**Time swing [%]**
Walking (1.1, 1.6)	1.14, 0.98	63, 60	37, 40
Climbing (asc., desc.)	1.2, 1.18	62.5, 62.7	37.5, 37.3
Stairs (asc., desc.)	1.41, 1.19	63.6, 61.2	36.4, 38.8
Jogging (2.6)	0.75	38	62
Running (4.0)	0.7	34	66
Cycling	1.0	100	–
Recovery	0.38	72.5	27.5
Squat Jump	1.1	38	62
Lifting (squat, stoop)	1.31, 1.22	100	–
Sit to Stand	2.25	100	–

*The total times describe the overall time of the analyzed biomechanical data in seconds. If applicable, a discrimination of stance phase and flight phase was made. Stance is considered to be when the analyzed leg is on the ground. Swing is considered to be when the analyzed leg has no ground contact. Stance and swing timing is provided as a relative value (in percent) of the total movement time. No swing phase is considered for cycling, lifting and the sit to stand transition*.

The absolute maxima were determined with the help of Matlab for the angle, the angular velocity, the angular acceleration, the moment and the power for all movements. Further, the mean absolute power was determined for all movements. Lastly, for a comparison of the maxima in stance and swing, the absolute maxima for stance and swing were determined for walking, running, climbing and stair ambulation. The mean and the standard deviation were calculated from these maxima (over all locomotion types). A Wilcoxon signed-rank test was used to test for a difference between mean values. A single asterisk (^*^p < 0.05) and a double asterisks (^**^p < 0.01) indicate a significant difference whereas non-significant differences are indicated by ns.

#### 2.3.2. Normalization of Angles

Digitized and calculated joint angles were zeroed to represent a hip, knee and ankle angle of zero degrees when in standing. Positive angles indicate hip and knee extension as well as ankle plantarflexion. Negative values indicate hip and knee flexion as well as ankle dorsiflexion. Based on the maximum joint angles for each movement, the range of motion was determined. The range of motion of the selected movements was compared to the mean range of motion based on an analysis of 30–40 years old males (approximately 96 individuals) that were free of any known impairments (Roaas and Andersson, 1982). The following paragraphs provide details of how angle data for each specific movement was adapted and standardized to allow for comparison and analysis.

##### 2.3.2.1. Walking, jogging and running

For a comparison of the range of motion of the level walking data, the mean (over one stride) angles of the hip, knee, and ankle were calculated from the 1.1 m/s level walking data (Lipfert, 2010; Grimmer and Seyfarth, 2014) and the level walking data provided as a reference in the climbing experiments (Lay et al., 2006). The level walking angles were shifted, to match the mean of the level walking angles from the climbing experiments, which were already standardized to standing. The same shift was applied to the 1.6 m/s walking data, the 2.6 m/s jogging data, and the 4.0 m/s running data.

##### 2.3.2.2. Climbing

The climbing angle data used Lay et al. (2006) was published in a way to have zero degree as neutral standing position. It was only necessary to invert the hip and knee angle to match our definition.

##### 2.3.2.3. Stairs

For the comparison of stair climbing and level walking in Riener et al. (2002), data from another gait laboratory, which was measured with a similar protocol, was used Frigo et al. (1998). Similar to the adaptation of the walking and running data, this data was shifted to match the mean joint angle of the level walking data from the climbing experiment. Prior to this, we had to invert the hip and knee angle to match our definition.

##### 2.3.2.4. Cycling

For cycling, the hip and the knee angle were inverted to match our definition. Further, 25° were subtracted from the hip angle as subjects were leaning their trunk forward by 20° to 30° (Ericson et al., 1986). Next, the ankle angle was normalized based on Sinclair et al. (2013), assuming a maximum plantarflexion of 3.95° compared to standing.

##### 2.3.2.5. Recovery, squat jump and lifting

For the recovery (Hsiao-Wecksler and Robinovitch, 2007), the squat jump (Mackala et al., 2013), and the lifting data (Hwang et al., 2009), all angles were inverted to match our definition. The data was provided in a way that we assumed zero degrees was achieved during upright standing.

##### 2.3.2.6. Sit to stand

As the final position of the sit to stand transitions is standing, the hip, knee, and ankle angle were adapted to be zero at the final time frame of the digitized data (Roebroeck et al., 1994).

#### 2.3.3. Specific Data Details

This subsection will provide further details regarding specific methodologies that were used for some movements to make the data comparable.

##### 2.3.3.1. Cycling

The time of one cycle of cycling was indirectly provided as subjects were instructed to cycle at 60 rpm. Thus, one repetition required 1 s.

##### 2.3.3.2. Recovery

Recovery movement data was analyzed for the young subject group at the 35% of body height horizontal stepping distance condition (starting in forward lean, subjects stepped to a target line located at this distance; Hsiao-Wecksler and Robinovitch, 2007). The data published covers a time frame of 1.5 s, and it was synchronized to the event of the foot contact of the recovery motion, which occurred at 0.5 s. The mean recovery movement duration, the time interval between tether release and the instant on the force plate, lasted 0.38 s. We therefore removed the first 0.12 s from the analysis. The 0.38 s are considered to be swing, and the following second was considered to be the stance phase.

##### 2.3.3.3. Squat jump

The jumping data was digitized and calculated only for the right leg. The movement time of the right leg was determined based on the provided flight time of 0.68 s. The ankle power during flight time was considered to be zero. Based on this assumption the flight phase required about 62% of the total movement time, and in combination with the additional 38% (0.42 s), a total of 1.1 s was assumed for the duration of this movement.

##### 2.3.3.4. Lifting

The times for the lifting movements squat and stoop were determined based on the provided peak power values of the hip. To calculate the joint power, the angular velocity had to be multiplied with the provided joint moment. To get the angular velocity, the provided joint angles had to be differentiated, where the required movement time was varied to achieve the angular velocity that results in the provided hip peak power. For the squat, the movement duration was 1.31 s and for the stoop, the duration was 1.22 s.

##### 2.3.3.5. Sit to stand

The provided angular velocity data for the sit to stand transition already included oscillations (especially ankle) (Roebroeck et al., 1994), which resulted in even higher oscillations when calculating the angular acceleration. We therefore decided not to use the angular velocity provided in the publication. Instead, we calculated both angular velocity and angular acceleration based on the provided angle data and the use of a filter that what well-suited for angular data.

## 3. Results

While the results of our analysis (e.g., maxima, ranges) can be found in Figures 1–6, the biomechanical parameters for the complete movement cycle (e.g., ankle angle during waling) are presented in the Appendix for the hip (Figure 7), the knee (Figure 8), and the ankle (Figure 9).

**Figure 1:**
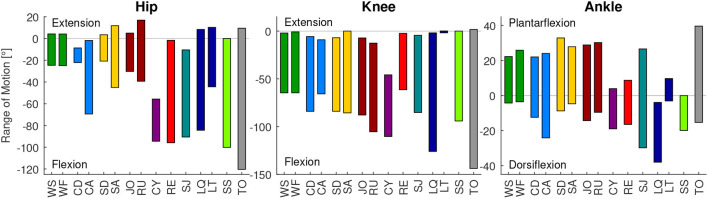
Range of motion of the analyzed movement tasks for the hip, knee, and ankle joint, compared to the mean total range of motion measurements for each joint (TO, gray) determined by Roaas and Andersson (1982). From left to right: walking slow (WS), walking fast (WF), climbing descend (CD), climbing ascend (CA), stair descend (SD), stair ascend (SA), jogging (JO), running (RU), cycling (CY), recovery (RE), squat jump (SJ), squat lifting (LQ), stoop lifting (LT), and sit to stand (SS).

**Figure 2:**
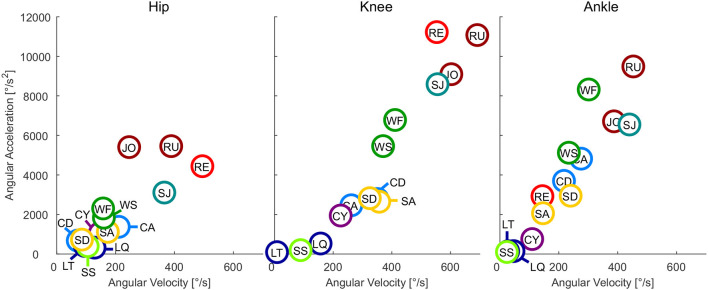
Maximum angular velocity vs. maximum angular acceleration of the hip, knee, and ankle for all analyzed movements. Walking slow (WS), walking fast (WF), climbing descend (CD), climbing ascend (CA), stair ascend (SA), stair descend (SD), jogging (JO), running (RU), cycling (CY), recovery (RE), squat jump (SJ), squat lifting (LQ), stoop lifting (LT), and sit to stand (SS).

**Figure 3:**
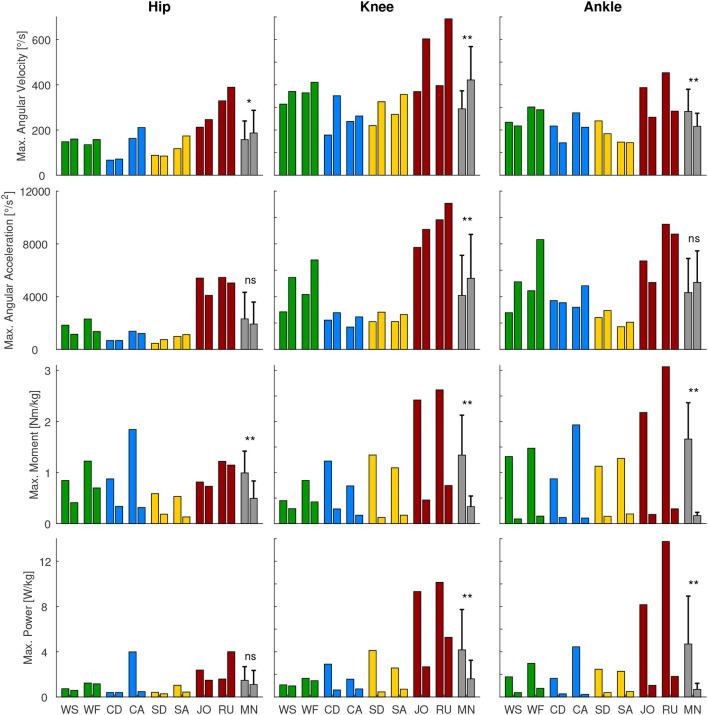
Maximum angular velocity, angular acceleration, moment, and power for the hip, knee, and ankle for the gait related movements [walking slow (WS), walking fast (WF), climbing descend (CD), climbing ascend (CA), stair descend (SD), stair ascend (SA), jogging (JO), and running (RU)]. For comparison of the maxima of stance (left bar) and swing phase (right bar), the mean over these conditions (MN, gray bars) and the statistical difference was determined. *p < 0.05, **p < 0.01, ns, non-significant.

**Figure 4:**
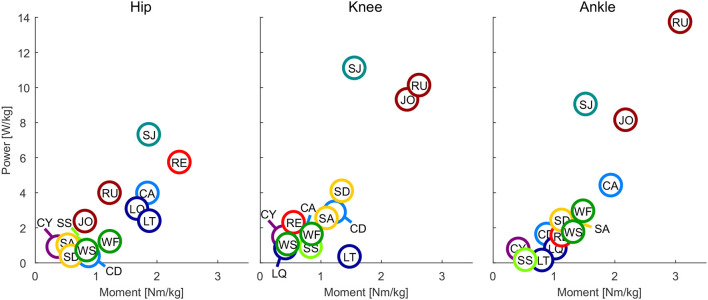
Maximum power vs. maximum moment of the hip, knee, and ankle for all analyzed movements. Walking slow (WS), walking fast (WF), climbing descend (CD), climbing ascend (CA), stair ascend (SA), stair descend (SD), jogging (JO), running (RU), cycling (CY), recovery (RE), squat jump (SJ), squat lifting (LQ), stoop lifting (LT), sit to stand (SS).

**Figure 5:**
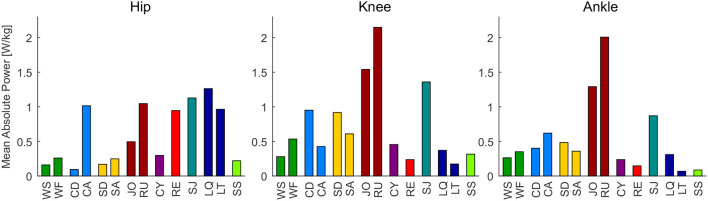
Mean absolute hip, knee and ankle power for the analyzed movements. From let to right: walking slow (WS), walking fast (WF), climbing descend (CD), climbing ascend (CA), stair descend (SD), stair ascend (SA), jogging (JO), running (RU), cycling (CY), recovery (RE), squat jump (SJ), squat lifting (LQ), stoop lifting (LT), sit to stand (SS).

**Figure 6:**
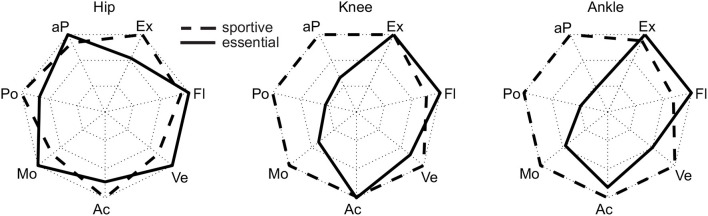
Normalized maximum requirements for the essential (solid) and the sportive (dashed) movement tasks. The normalization was performed such that the maximum (sportive or essential) becomes 1, which represents the outer limit of the radar plot. The parameters are the maximum joint extension (Ex, at ankle plantarflexion), the maximum joint flexion (Fl, at ankle dorsiflexion), the maximum absolute angular velocity (Ve), the maximum absolute angular acceleration (Ac), the maximum absolute moment (Mo), the maximum absolute power (Po) and the maximum absolute average power (aP).

**Figure 7:**
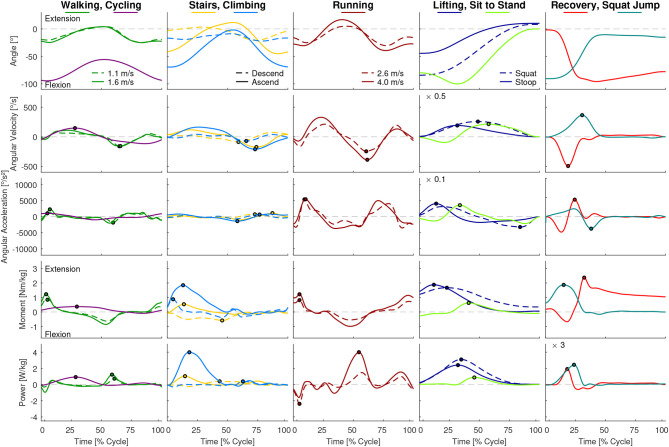
Hip angle, angular velocity, angular acceleration, moment and power for all analyzed movements. Dots indicate the maxima used for Figures 2, 4. For visibility some of the graphs were scaled with a factor, which is located at the top left of these graphs. For example, a scaling factor of 0.1 indicates that the y-axis must be multiplied by 0.1.

**Figure 8:**
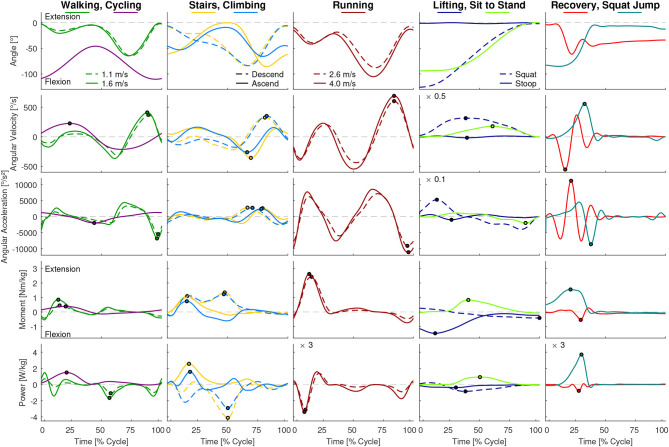
Knee angle, angular velocity, angular acceleration, moment and power for all analyzed movements. Dots indicate the maxima used for Figures 2, 4. For visibility some of the graphs were scaled with a factor, which is located at the top left of these graphs. For example, a scaling factor of 0.1 indicates that the y-axis must be multiplied by 0.1.

**Figure 9:**
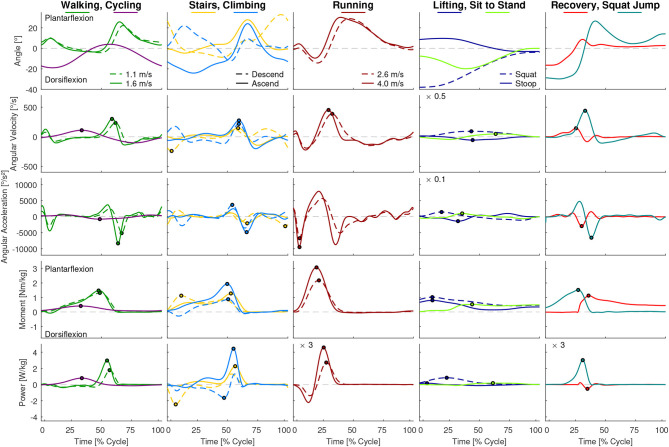
Ankle angle, angular velocity, angular acceleration, moment and power for all analyzed movements. Dots indicate the maxima used for Figures 2, 4. For visibility some of the graphs were scaled with a factor, which is located at the top left of these graphs. For example, a scaling factor of 0.1 indicates that the y-axis must be multiplied by 0.1.

### 3.1. Range of Motion

The mean total range of motion of humans (TO) was found to be 9° to −120° degree for the hip, 2° to 144° for the knee, and 40° to −15° for the ankle (Figure 1). The movements that require the largest hip range of motion are the sit to stand transition, recovery and squat lifting. The largest hip extension is required for running, while the largest hip flexion is required for the sit to stand transition. The largest knee range of motion is required for the squat lifting, the sit to stand transition and running. While most movement tasks require almost full knee extension, the maximum flexion was achieved for squat lifting. The maximum ankle range of motion is required for the squat jump, while stair descending requires the largest plantarflexion, squat lifting requires the largest dorsiflexion.

At the hip and the knee, the mean total range of motion of humans (TO) was almost covered by the analyzed movement tasks. However, a larger ankle range of motion was identified for the movement tasks, compared to the total range measurement (TO, Figure 1).

The identified range of motion of the analyzed movements represents the mean of the analyzed populations. Greater magnitudes and ranges were achieved for individual subjects. Based on the maximum range of motion measurements of Roaas and Andersson (1982), the maximum extension and flexion of single subjects were 35° and −150° for the hip, 10° to −160° for the knee, and 55° (plantarflexion) to −40° (dorsiflexion) for the ankle.

### 3.2. Joint Angular Velocity and Acceleration

At the hip, the highest angular velocity was found for recovery and the highest angular acceleration was found in running and jogging (Figure 2). At the knee, the highest angular velocity was identified for running and the highest angular acceleration for recovery. At the ankle both angular velocity and acceleration were highest for running.

We analyzed the gait-related movement tasks (walking, running, stairs, climbing) on the difference of maximum angular velocity and acceleration during stance and swing phase. We found that for all joint, except for the ankle angular velocity, the mean is equal or higher during swing phase compared to stance (Figure 3).

### 3.3. Joint Moment and Power

At the hip, the maximum joint moment was found for recovery, while the maximum power was found for the squat jump (Figure 4). The squat jump was also identified to require the maximum knee power. Running and jogging were found to require the highest knee and ankle moment. The highest power at the ankle is required in running.

When analyzing the gait related movement tasks (walking, running, stairs, climbing) on the difference of maximum moment and power during stance and swing phase, we found that all conditions and joints require higher moments during stance (Figure 3). Regarding maximum power, no difference was found for the hip, whereas for the knee and the ankle the stance required higher maximum power than the swing phase.

When analyzing the mean absolute power of the hip (Figure 5), we found that sportive movements, such as running and the squat jump, require similar levels (1 W/kg) as essential movements such as climbing (ascends), lifting and recovery. At the knee and the ankle, running, jogging and the squat jump had the highest mean absolute power requirements with up to 2.1 W/kg, while essential movements achieved up to 1 W/kg for the knee and up to 0.6 W/kg at the ankle.

## 4. Discussion

To the knowledge of the authors, this is the first comprehensive analysis of sagittal lower limb biomechanics for a variety of movement tasks. Our aim was to identify the most demanding movements and to extract recommendations for the dimensioning of drivetrains for wearable lower limb robotics and humanoid robots that are capable of human-like movements. The following subsections will first discuss the findings of the specific biomechanical parameters (e.g., range of motion, maximum angular velocity, etc.) and the implications regarding the design requirements for stance and swing. The identified maxima of the biomechanical quantities of the essential daily tasks are then compared to maximum performance values of athletes. These values can enable engineers to design robotic lower limbs with greater performance or with specifications above the normal human movement capabilities. As mimicking human lower limb joint behavior with robots is challenging, we briefly propose mechanical concepts for reducing requirements.

### 4.1. Range of Motion

We found that the presented mean maximal human ankle dorsiflexion value from Roaas and Andersson (1982) was 15° to 23° less compared to the dorsiflexion during squat jump and squat lifting (Figure 1). Slightly smaller differences occur when compared to maximal values based on another study (Moromizato et al., 2016). We believe that a part of this difference could result from the definition of the neutral position (subject supine with knee in 45° flexion) used in Roaas and Andersson (1982) and Moromizato et al. (2016), compared to our approach that normalized the data to standing. Further, increased dorsiflexion at the ankle could be possible with increased knee flexion due to limitations in maximum muscle length. While up to 126° of knee flexion were achieved in squat lifting, the dorsiflexion of the ankle was measured with a knee flexion of 45° in Roaas and Andersson (1982). Based on previous literature, we found that a limitation due to muscle length is less likely, as a knee flexion angle of even 20° will avoid muscle length limitations (Baumbach et al., 2014). A more plausible reason for increased dorsiflexion during the squat jump and squat lifting could be that the Achilles tendon and the calf muscles are stretched by the external load at the beginning of both movements (see plantarflexion moments in the Appendix). Thus, we believe that the applied joint moments can cause an increased range of motion, compared to the passive range of motion measurements (Roaas and Andersson, 1982; Moromizato et al., 2016) without larger applied joint moments.

Based on our findings, essential daily movements, such as lifting or sit to stand transitions, require almost the full human hip and knee range of motion. Increased requirements, compared to the passive measurements, are required for the ankle dorsiflexion. Thus, we recommend to consider the full motion range of hip (17° to −120°) and knee (2° to −144°) for the design of wearable robotic limbs or human-like robots. This similarly applies to ankle plantarflexion (40°), while an extended range of motion needs to be taken into account for the ankle dorsiflexion (−38°).

When comparing essential and sportive movements, there is no clear difference in the RoM regarding joint extension and flexion (Figure 6). Thus, we would not make a difference when designing a robotic actuator for one or the other.

### 4.2. Angular Velocity and Acceleration

As expected, the sportive movements of running, jogging, and the squat jump resulted in the maximum angular velocities and accelerations throughout the complete movement cycle. Additionally, recovery achieved comparable results at the hip and the knee, but not for the ankle. When designing lower limb wearable or humanoid robots, the sportive tasks of running, jogging and the squat jump may not need to be considered. However, we believe that the recovery movement should be designed into the range of feasible movements. In doing so, anthropomorphic robots could benefit from a fast reactive human-like safety mechanism that could prevent falling. If artificial robotic joints work in cooperation with the human lower limb, as can be found in prostheses, it is necessary to enable the wearable robot to achieve the maximum angular velocity and acceleration that was found during recovery in this study. For example, while the hip and knee of a transtibial amputee try to recover from a tripping event, an artificial powered prosthetic foot with limited angular velocity and acceleration may not be able to dorsiflex fast enough to provide ground clearance. In order to sufficiently enable function for daily life, maximum angular velocities of 500°/s for the hip, 550°/s for the knee and 300°/s for the ankle seem appropriate. The angular acceleration should be 4,400°/s^2^, 11,200°/s^2^, and 8,300°/s^2^ for the hip, knee, and ankle, respectively. To perform sportive movements, increasing the maximum velocity is recommended for the knee and the ankle, and increased maximum angular acceleration is recommended for the hip and ankle (Figure 6).

### 4.3. Moment and Power

This study found that, similar to the angular velocity and acceleration, maximum moment and power are required to perform the sportive movement tasks of running, jogging and the squat jump. The least difference to the essential movements was found at the hip joint, where recovery had the highest requirements from the essential tasks. Increased moment and power requirements, as well as increased requirements in angular velocity and acceleration, were not completely unexpected for recovery and squat jump, as both were the only movements where subjects perform at their physiological limits. To provide the capabilities for daily life, a maximum moment of 2.4 Nm/kg for the hip, 1.5 Nm/kg for the knee and 1.9 Nm/kg for the ankle seem appropriate. A maximum power of 5.8 W/kg, 4.1 W/kg, and 4.3 W/kg appears recommendable for the hip, knee, and ankle, respectively. For sportive movements, the more distal the joint, the more the requirements increase with up to 3.1 Nm/kg and 13.8 W/kg at the ankle.

Next to the peak power, which defines the maximum possible power of the actuator, the mean absolute power was analyzed. This can help to select the maximum continuous power of an actuator and to define heat dissipation requirements. At the knee and the ankle, the sportive movements required up to twice the mean absolute power of the essential movements (Figure 6). In comparison, lifting, recovery and ascending climbing have similar requirements at the hip as running and the squat jump. We would not expect that the squat jump or recovery are tasks that will be performed continuously. Thus, their contributions to heating will be less. Also, lifting is a task we would not consider to be performed continuously, however, some specific activities may require lifting over longer periods of time. Zero to fifty eight minutes of daily forward bending time was identified for those working in industrial jobs (Lagersted-Olsen et al., 2016). Most critical for defining design requirements for continuous power are running (hip, knee, ankle), jogging (knee, ankle) and climbing ascent (hip). In conclusion, while the hip specifications are defined by the essential movements, the knee and ankle specifications could be reduced to half when not including the sportive tasks. As continuous climbing (ascend or descend) is a valid scenario it would be ideal to cover a mean absolute power of 1 W/kg at the hip and the knee, and 0.6 W/kg at the ankle.

### 4.4. Differences in Performance in Stance and Swing

We expected that the maximum joint requirements will differ for the stance and the swing phase within locomotion tasks. Our hypothesis that increased angular velocity and acceleration are required in the non-weight bearing swing was only confirmed for the knee. In the hip, only the angular velocity was larger in swing, while for the hip and the ankle, the angular accelerations were not different. In contrast to our overall assumption, the maximum angular velocity was highest in stance at the ankle, which was achieved during push-off in late stance where the combined shortening of the calf muscle fibers and the Achilles tendon lead to the observed behavior (Ishikawa et al., 2007).

For the maximum moment and maximum power, we expected increased requirements in weight bearing stance. This hypothesis was confirmed for all but the hip maximum power, where no difference was identified. Based on the findings for the maximum kinematic and kinetic values, we believe that it is possible to use actuators with smaller power and moment specifications for the swing phase. In contrast, we do not believe that it is an advantage to use an actuator with less maximum acceleration or maximum velocity during stance as the swing phase determines the required maximum specification for most conditions.

### 4.5. Human Lower Limb Joint Performance Limits

While this study focuses on the lower limb joint requirements for movements of daily life, other movements or increased speeds and loads can require increased capabilities. To investigate kinematic-kinetic relations without being specific to a movement, researchers have used dynamometers. It has been shown that with increasing angular velocity, the maximum possible joint moment is reduced, and that at certain joint angles, the highest moments can be achieved (Anderson et al., 2007). For young males (non-athletes), the identified maximum isometric extension and flexion moment for the hip were 2.8 Nm/kg and 1.9 Nm/kg, for the knee were 2.8 Nm/kg and 1.5 Nm/kg, and for the ankle were 1.6 Nm/kg (plantarflexion) and 0.6 Nm/kg (dorsiflexion). While the identified maximum hip moment is not achieved in the analyzed daily life movements, the maximum knee moment is achieved, and the maximum ankle moment found in 4 m/s running is larger than the values achieved with the dynamometer. While the values provided in Anderson et al. (2007) are from an isometric condition, eccentric movements are able to achieve about 150% (at knee measured in athletes) of the isometric moment performance (Yeadon et al., 2006). During running, this increase in performance is explainable because the maximum moment occurs at maximum dorsiflexion, which is achieved by an eccentric muscle behavior (Ishikawa et al., 2007).

Maximum knee angular velocity was identified to be 768°/s and 1535°/s for two athletes (high jump, material arts, Yeadon et al., 2006). In sprint running of national level sprinters and middle distance runners (9.7 m/s and 8.9 m/s), maximum angular velocities of 570°/s and 584°/s were achieved at the hip, 970°/s and 425°/s at the knee, and 930°/s and 960°/s at the ankle (Belli et al., 2002; Zhong et al., 2017). While Zhong et al. (2017) analyzed the whole running cycle, Belli et al. (2002) only analyzed the stance, which explains the low value at the knee. Based on this athlete data, the maxima of the provided movements is about 300% of the daily requirements (hip: 500°/s, knee: 550°/s, ankle: 300°/s) for the knee and the ankle whereas the hip maximum angular velocity is just slightly increased. If this finding is true, it would mean that the maximum velocity of the human hip is nearer to its performance limits during our selection of essential movements compared to the knee and ankle.

At 8.9 m/s running, the maximum moment during stance was found to be 3.4 Nm/kg at the hip, 3.9 Nm/kg at the knee, and 3.4 Nm/kg at the ankle (Belli et al., 2002). While running at 4 m/s almost achieved this moment at the ankle, maximum sprinting moments are higher compared to those achieved during the essential movements (hip: 2.4 Nm/kg, knee: 1.5 Nm/kg, ankle: 1.9 Nm/kg). Even higher maximum extension moments were achieved by athletes during the long jump, where the hip, knee and ankle achieved maximum extension moments of 8.4 Nm/kg, 3.9 Nm/kg and 5.2 Nm/kg, respectively (Stefanyshyn and Nigg, 1998). A second study on the long jump confirmed these results (Muraki et al., 2008). One could expect even larger extensor moments are required for weight lifting of 100 kg, but experimental data revealed that peak extension moments were about 2.7 Nm/kg for the hip, 1.5 Nm/kg for the knee, and 2.5 Nm/kg for the ankle (moments only normalized by human mass, Kipp et al., 2011). Compared to the maximum angular velocity, maximum joint moments of athletes are 260–350% of the requirements for the essential movements.

In sprinting, a maximum of 53 W/kg was required for hip extension, 19 W/kg for braking the knee in late swing and 66 W/kg for absorbing energy in early stance at the ankle (Zhong et al., 2017). When only analyzing the stance in Belli et al. (2002), maximum propulsion in late stance was found to be 26 W/kg for hip extension, 22 W/kg for knee extension, and 40 W/kg for ankle plantarflexion. While both studies differ in the results, maximum joint power during sprinting was found to be much higher when compared to the maxima identified for the essential daily tasks (hip: 5.8 W/kg, knee: 4.1 W/kg, ankle: 4.3 W/kg). Similar values were identified for the long jump (Stefanyshyn and Nigg, 1998; Muraki et al., 2008), while the negative knee power during stance is greater than 50 W/kg. When comparing the study of Zhong et al. (2017) to the requirements of the essential tasks, the hip power is increased by about 900%, the knee power by about 460%, and the ankle power by about 1,500%. These large increases in the maximum power highlight that the increases in maximum velocity and maximum acceleration are amplified.

When summarizing the comparison of the requirements for the essential movement tasks and the maximum joint performance in athletes, it becomes clear that matching athlete-like joint performance with robots is much more challenging compared to providing solutions for the essential tasks. Presently, in order to mimic the essential tasks with human-like robots, robotic actuators require biologically-inspired mechanisms such as elastic structures to reduce actuator requirements (Au et al., 2009; Grimmer et al., 2016). Since we believe that achieving athlete-like performance also benefits from biologically-inspired mechanisms, we briefly propose options to reduce the joint performance requirements subsequently.

### 4.6. Reducing Actuator Requirements

Human-like robots and wearable lower limb robotics primarily use motors for actuation (Yan et al., 2015; Torricelli et al., 2016; Windrich et al., 2016), though hydraulic and pneumatic solutions have also been investigated (Versluys et al., 2009; Kuindersma et al., 2016; del Carmen Sanchez-Villamañan et al., 2019). To reduce the actuator requirements, such as the acceleration, velocity, moment, and power, various mechanical alternatives or mechanical extensions have been investigated for humanoid robots (Tagliamonte et al., 2012; Torricelli et al., 2016), exoskeletons (del Carmen Sanchez-Villamañan et al., 2019), and prostheses (Grimmer and Seyfarth, 2014; Pieringer et al., 2017). It was found that springs in series with a motor can reduce the maximum angular velocity and acceleration requirements for various gaits and speeds, which will also result in reduced maximum power requirements (Paluska and Herr, 2006; Grimmer et al., 2014, 2016; del Carmen Sanchez-Villamañan et al., 2019). With reduced maximum power requirements for the motor, the necessary energy requirements can also be reduced. Alternatively, parallel springs are able to reduce the maximum moment requirements of a motor (Yang et al., 2008; Grimmer et al., 2012; Häufle et al., 2012) due to compensating for static loads (Beckerle et al., 2017). Combinations of series and parallel springs (Grimmer et al., 2012; Mathijssen et al., 2014), as well as unidirectional parallel springs (Au et al., 2009; Sup et al., 2009; Eslamy et al., 2012), can provide combined effects. Variable spring stiffness was proposed to improve the efficiency for changing conditions (Tagliamonte et al., 2012; Vanderborght et al., 2013). In addition to springs, dampers have been used to mimic negative joint work, as can be found during the swing phase of the knee in most gaits (Seroussi et al., 1996; Johansson et al., 2005; Grimmer and Seyfarth, 2014; Sohn et al., 2018) or at the ankle during descending stairs (Riener et al., 2002; Eslamy et al., 2013). Clutches are another option that can be used to reduce joint requirements (Plooij et al., 2015). They are used to store energy in springs and to release the energy when required (Häufle et al., 2012; Cherelle et al., 2016). Clutches can also unload the motor of a series elastic actuator during phases where no length adaptation is required.

### 4.7. Requirements in Different Robotic Applications

Depending on the robotic application, the target outputs of the joint specific actuators can differ from the identified human biomechanics. To mimic human like movement behavior, the kinematic measures (RoM, angular velocity, angular acceleration) have to be imitated by humanoid robots, exoskeletons and prostheses. Compared, kinetics (moment, power) could be different based on the robotic application. An exoskeleton that should be used for rehabilitation for those without walking capabilities has to carry itself and the user. Thus, the moment and power requirements for the exoskeleton would be higher compared to the human joint requirements. Depending on the inertia of each exoskeleton segment, the increases might not scale with the human requirements. In contrast, an exoskeleton that should only assist a user without any mobility impairments could be designed to achieve much smaller kinetic target outputs. An actuator with 12 Nm of peak torque was able to reduce the metabolic cost of walking by 21% for subjects, when walking with a hip exoskeleton (Lee et al., 2017). Lower target outputs would be also possible for prostheses or humanoid robots with reduced total or segment inertia compared to humans. So far, typically masses are above human thresholds. For example, the Atlas-unplugged has a mass of about 180 kg at a body height of 188 cm (Nelson et al., 2019). Our comprehensive overview could also be a great source to determine the requirements of these robots, which have different mass distribution than humans. The summarized kinematics could be used in combination with a robotic model (including segment properties) to estimate the required joint moment and joint power values.

For applying the findings of this work to real-world design, we recommend that system designers identify the most demanding movement tasks that are envisioned for their application and target population. Subsequently, technical solutions, such as those introduced herein, can be used to realize the desired movements efficiently.

### 4.8. Methodological Considerations

While the squat jump data was from semi-professional athletes, all other data was from non-athlete populations. Thus, the squat jump data may be exaggerated, compared to the other movements, due to the population.

Be aware, due to digitizing and filtering of most of the movement data, the shape and the maxima of all the data might not represent the exact values identified in the data source. Furthermore, as part of the data was calculated based on the digitized values (e.g., angular acceleration, power), differences may propagate to these quantities as well.

While the methods we used to prepare the data can result in a variation of the values, also the different methods used by the original sources can lead to less comparable data. In Table 1 we summarized published information on the experimental setup and analysis procedures. Variations in data can occur due to 2D or 3D kinematic data recordings, calculated angles in the sagittal or limb plane, different filtering procedures, different measurement frequencies, different methods (inputs and segment properties) to compute joint moments based on inverse dynamics, and different numbers of repetitions as well as subject selection (number, characteristics). Small variations in 2D compared to 3D based angles were found for movement task with fixed feet (squat, cycling, García-López and del Blanco, 2017; Schurr et al., 2017) as well as for a locomotion task with flight phases (race walking, Hanley et al., 2018). Differences exist due to the 2D camera position compared to the marker positions (difference in height and left/right) and due to joint movements (mainly hip internal or external rotation and adduction or abduction) in the sagittal and longitudinal axes. The second issue will also occur when calculating the angles in the sagittal plane instead of using the limb plane defined by the hip, the knee, and the foot.

Low pass filters, as used for human kinematics and kinetics will reduce signal noise but also the amplitude of signal peaks (Winter et al., 1974). A similar effect occurs when determining the mean, based on multiple subject repetitions, and following based on multiple subject means. In consequence, we believe that the presented values might underestimate especially the maximum acceleration of the movement tasks. Single subjects will have increased values. If provided, standard deviations of the included studies could be checked to get additional information on it.

The human movement studies used different measurement frequencies (Figure 1) to analyze the kinematics and kinetics. Winter et al. (1974) showed that toe marker acceleration and knee angular velocity during walking can be sufficiently determined at a measurement frequency of 60 Hz. The lowest frequency used to record the original data included in this work was 40 Hz, for the sit to stand transition. As the total time (Table 2) of this transition is double of the walking stride time and it is not a rapid movement, it should be sufficient to record such data without losing characteristic information.

All studies used inverse dynamics to compute the joint moments. Different methodological approaches such as the inclusion of wobbling masses (Günther et al., 2003) or different segment properties (mass, length, center of gravity, moments of inertia, Winter, 2009) will influence the results.

For an improved comparison of such movement data, the ideal approach would be to have the same subjects performing all movement tasks followed by an analysis with a single method (Wojtusch and von Stryk, 2015; Mandery et al., 2016).

## 5. Conclusion

With this study we provide a novel comprehensive overview of human lower limb biomechanics for daily essential and sportive movements. The data was extracted to improve the dimensioning of robotic limbs that target human-like performance. Within the analysis we found that system designers should consider the full human range of motion for all lower limb joints, with an extended range of motion for ankle dorsiflexion. For most of the analyzed biomechanical features, the hip specifications for the essential movements are close to those for the sportive movements, whereas sportive movements require increased capabilities at the human knee and ankle. For locomotion tasks, it does not seem worthwhile to use different technical solutions to realize the angular velocity and angular acceleration throughout stance and swing. In contrast, different designs can be considered to match the necessary human joint moment and power during stance and swing. While designers already take advantage of biologically-inspired mechanisms, e.g., springs mimicking tendon functionality in cyclic essential movements, such approaches appear to be even necessary to reach the performance of sportive movements, especially when targeting athlete-like performance or augmented performance above natural human capability. Beyond these insights, we expect the broad analysis of human movements in this work to serve as a data base for engineering future generations of anthropomorphic robots.

## Data Availability Statement

All datasets generated for this study are included in the article/Supplementary Material. Part of the data is also available in the original literature sources.

## Author Contributions

The study concept was developed by MG and PB. The literature research, data extraction, and the data analysis were performed by MG and AE. Interpretation was performed by all authors. MG was responsible for drafting the article. All authors revised the article and provided approval for publication of the content. All authors agreed to be accountable for all aspects of the work in ensuring that questions related to the accuracy or integrity of any part of the work are appropriately investigated and resolved.

### Conflict of Interest

The authors declare that the research was conducted in the absence of any commercial or financial relationships that could be construed as a potential conflict of interest.

## References

[B1] Anderson D. E. Madigan M. L. Nussbaum M. A. (2007). Maximum voluntary joint torque as a function of joint angle and angular velocity: model development and application to the lower limb. J. Biomech. 40, 3105–3113. 10.1016/j.jbiomech.2007.03.022 17485097 PMC6820133

[B2] Au S. K. Weber J. Herr H. (2009). Powered ankle–foot prosthesis improves walking metabolic economy. IEEE Trans. Robot. 25, 51–66. 10.1109/TRO.2008.2008747

[B3] Baumbach S. F. Brumann M. Binder J. Mutschler W. Regauer M. Polzer H. (2014). The influence of knee position on ankle dorsiflexion-a biometric study. BMC Musculoskelet. Disord. 15:246. 10.1186/1471-2474-15-246 25053374 PMC4118219

[B4] Beckerle P. Verstraten T. Mathijssen G. Furnémont R. Vanderborght B. Lefeber D. (2017). Series and parallel elastic actuation: influence of operating positions on design and control. IEEE/ASME Trans. Mechatr. 22, 521–529. 10.1109/TMECH.2016.2621062

[B5] Belli A. Kyröläinen H. Komi P. (2002). Moment and power of lower limb joints in running. Int. J. Sports Med. 23, 136–141. 10.1055/s-2002-20136 11842362

[B6] Bratt Å. Ericson M. O. (1985). Biomechanical Model for Calculation of Joint Loads During Ergometer Cycling. Stockholm: Royal Institute of Technology, Department of Mechanics.

[B7] Cappozzo A. Leo T. Pedotti A. (1975). A general computing method for the analysis of human locomotion. J. Biomech. 8, 307–320. 10.1016/0021-9290(75)90083-4 1184602

[B8] Chen B. Zi B. Wang Z. Qin L. Liao W.-H. (2019). Knee exoskeletons for gait rehabilitation and human performance augmentation: a state-of-the-art. Mech. Mach. Theory 134, 499–511. 10.1016/j.mechmachtheory.2019.01.016

[B9] Cherelle P. Grosu V. Cestari M. Vanderborght B. Lefeber D. (2016). The amp-foot 3, new generation propulsive prosthetic feet with explosive motion characteristics: design and validation. Biomed. Eng. Online 15:145. 10.1186/s12938-016-0285-8 28105954 PMC5249021

[B10] D'amico M. Ferrigno G. (1990). Technique for the evaluation of derivatives from noisy biomechanical displacement data using a model-based bandwidth-selection procedure. Med. Biol. Eng. Comput. 28, 407–415. 10.1007/BF02441963 2277540

[B11] del Carmen Sanchez-Villamañan M. Gonzalez-Vargas J. Torricelli D. Moreno J. C. Pons J. L. (2019). Compliant lower limb exoskeletons: a comprehensive review on mechanical design principles. J. Neuroeng. Rehabil. 16:55 10.1186/s12984-019-0517-9 31072370 PMC6506961

[B12] Do M. Chang V. Kuran N. Thompson W. (2015). Fall-related injuries among canadian seniors, 2005–2013: an analysis of the canadian community health survey. Health Promot. Chron. Dis. Prevent. Canada Res. Policy Pract. 35:99. 10.24095/hpcdp.35.7.01 26378768 PMC4910457

[B13] Drouin J. M. Valovich-mcLeod T. C. Shultz S. J. Gansneder B. M. Perrin D. H. (2004). Reliability and validity of the biodex system 3 pro isokinetic dynamometer velocity, torque and position measurements. Eur. J. Appl. Physiol. 91, 22–29. 10.1007/s00421-003-0933-0 14508689

[B14] Ericson M. O. Nisell R. Arborelius U. P. Ekholm J. (1986). Power output and work in different muscle groups during ergometer cycling. Eur. J. Appl. Physiol. Occupat. Physiol. 55, 229–235. 10.1007/BF02343792 3732250

[B15] Eslamy M. Grimmer M. Rinderknecht S. Seyfarth A. (2013). Does it pay to have a damper in a powered ankle prosthesis? a power-energy perspective, in IEEE 13th International Conference on Rehabilitation Robotics (ICORR) (Seattle, WA: IEEE), 1–8. 10.1109/ICORR.2013.6650362 24187181

[B16] Eslamy M. Grimmer M. Seyfarth A. (2012). Effects of unidirectional parallel springs on required peak power and energy in powered prosthetic ankles: Comparison between different active actuation concepts, in IEEE International Conference on Robotics and Biomimetics (ROBIO) (Guangzhou: IEEE), 2406–2412.

[B17] Ferrigno G. Pedotti A. (1985). Elite: a digital dedicated hardware system for movement analysis via real-time tv signal processing. IEEE Trans. Biomed. Eng. 32, 943–950. 10.1109/TBME.1985.325627 3905583

[B18] Frigo C. Rabuffetti M. Kerrigan D. Deming L. Pedotti A. (1998). Functionally oriented and clinically feasible quantitative gait analysis method. Med. Biol. Eng. Comput. 36, 179–185. 10.1007/BF02510740 9684457

[B19] García-López J. del Blanco P. A. (2017). Kinematic analysis of bicycle pedalling using 2d and 3d motion capture systems. ISBS Proc. Arch. 35:125 Available online at: https://commons.nmu.edu/isbs/vol35/iss1/125

[B20] Góngora Alonso S. Hamrioui S. de la Torre Díez I. Motta Cruz E. López-Coronado M. Franco M. (2019). Social robots for people with aging and dementia: a systematic review of literature. Telemed. e-Health 25, 533–540. 10.1089/tmj.2018.0051 30136901

[B21] Grimmer M. Eslamy M. Gliech S. Seyfarth A. (2012). A comparison of parallel-and series elastic elements in an actuator for mimicking human ankle joint in walking and running, in IEEE International Conference on Robotics and Automation (ICRA) (Saint Paul, MN: IEEE), 2463–2470.

[B22] Grimmer M. Eslamy M. Seyfarth A. (2014). Energetic and peak power advantages of series elastic actuators in an actuated prosthetic leg for walking and running. Actuators 3, 1–19. 10.3390/act3010001

[B23] Grimmer M. Holgate M. Holgate R. Boehler A. Ward J. Hollander K.. (2016). A powered prosthetic ankle joint for walking and running. Biomed. Eng. Online 15:37. 10.1186/s12938-016-0286-7 28105953 PMC5249039

[B24] Grimmer M. Quinlivan B. T. Lee S. Malcolm P. Rossi D. M. Siviy C.. (2019a). Comparison of the human-exosuit interaction using ankle moment and ankle positive power inspired walking assistance. J. Biomech. 83, 76–84. 10.1016/j.jbiomech.2018.11.023 30514626 PMC6375290

[B25] Grimmer M. Riener R. Walsh C. J. Seyfarth A. (2019b). Mobility related physical and functional losses due to aging and disease-a motivation for lower limb exoskeletons. J. Neuroeng. Rehabil. 16:2. 10.1186/s12984-018-0458-8 30606194 PMC6318939

[B26] Grimmer M. Seyfarth A. (2014). Mimicking human-like leg function in prosthetic limbs, in Neuro-Robotics, ed. Artemiadis P. (Dordrecht: Springer), 105–155.

[B27] Günther M. Sholukha V. A. Kessler D. Wank V. Blickhan R. (2003). Dealing with skin motion and wobbling masses in inverse dynamics. J. Mech. Med. Biol. 3, 309–335. 10.1142/S0219519403000831

[B28] Hanley B. Tucker C. B. Bissas A. (2018). Differences between motion capture and video analysis systems in calculating knee angles in elite-standard race walking. J. Sports Sci. 36, 1250–1255. 10.1080/02640414.2017.1372928 28850306

[B29] Häufle D. F. Taylor M. Schmitt S. Geyer H. (2012). A clutched parallel elastic actuator concept: towards energy efficient powered legs in prosthetics and robotics, in 2012 4th IEEE RAS & EMBS International Conference on Biomedical Robotics and Biomechatronics (BioRob) (Rome: IEEE), 1614–1619.

[B30] Hof A. (2000). On the interpretation of the support moment. Gait Post. 12, 196–199. 10.1016/S0966-6362(00)00084-9 11154929

[B31] Hsiao-Wecksler E. T. Robinovitch S. N. (2007). The effect of step length on young and elderly women's ability to recover balance. Clin. Biomech. 22, 574–580. 10.1016/j.clinbiomech.2007.01.013 17391819

[B32] Hwang S. Kim Y. Kim Y. (2009). Lower extremity joint kinetics and lumbar curvature during squat and stoop lifting. BMC Musculoskelet. Disord. 10:15. 10.1186/1471-2474-10-15 19183507 PMC2651112

[B33] Ishikawa M. Pakaslahti J. Komi P. (2007). Medial gastrocnemius muscle behavior during human running and walking. Gait Post. 25, 380–384. 10.1016/j.gaitpost.2006.05.002 16784858

[B34] Johansson J. L. Sherrill D. M. Riley P. O. Bonato P. Herr H. (2005). A clinical comparison of variable-damping and mechanically passive prosthetic knee devices. Am. J. Phys. Med. Rehabil. 84, 563–575. 10.1097/01.phm.0000174665.74933.0b 16034225

[B35] Kapsalyamov A. Jamwal P. K. Hussain S. Ghayesh M. H. (2019). State of the art lower limb robotic exoskeletons for elderly assistance. IEEE Access 7, 95075–95086. 10.1109/ACCESS.2019.2928010

[B36] Kipp K. Harris C. Sabick M. B. (2011). Lower extremity biomechanics during weightlifting exercise vary across joint and load. J. Strength Condit. Res. 25, 1229–1234. 10.1519/JSC.0b013e3181da780b 21240030

[B37] Kuindersma S. Deits R. Fallon M. Valenzuela A. Dai H. Permenter F. (2016). Optimization-based locomotion planning, estimation, and control design for the atlas humanoid robot. Auton. Robots 40, 429–455. 10.1007/s10514-015-9479-3

[B38] Lagersted-Olsen J. Thomsen B. L. Holtermann A. Søgaard K. Jørgensen M. B. (2016). Does objectively measured daily duration of forward bending predict development and aggravation of low-back pain? A prospective study. Scand. J. Work Environ. Health. 42, 528–537. 10.5271/sjweh.3591 27606607

[B39] Larsen G. E. George J. D. Alexander J. L. Fellingham G. W. Aldana S. G. Parcell A. C. (2002). Prediction of maximum oxygen consumption from walking, jogging, or running. Res. Q. Exerc. Sport 73, 66–72. 10.1080/02701367.2002.10608993 11926486

[B40] Lay A. N. Hass C. J. Gregor R. J. (2006). The effects of sloped surfaces on locomotion: a kinematic and kinetic analysis. J. Biomech. 39, 1621–1628. 10.1016/j.jbiomech.2005.05.005 15990102

[B41] Lee J. Seo K. Lim B. Jang J. Kim K. Choi H. (2017). Effects of assistance timing on metabolic cost, assistance power, and gait parameters for a hip-type exoskeleton, in 2017 International Conference on Rehabilitation Robotics (ICORR) (London: IEEE), 498–504. 10.1109/ICORR.2017.8009297 28813869

[B42] Lipfert S. W. (2010). Kinematic and Dynamic Similarities Between Walking and Running. Hamburg: Kovač.

[B43] Mackala K. Stodólka J. Siemienski A. Coh M. (2013). Biomechanical analysis of squat jump and countermovement jump from varying starting positions. J. Strength Condit. Res. 27, 2650–2661. 10.1519/JSC.0b013e31828909ec 23552341

[B44] Mandery C. Terlemez Ö. Do M. Vahrenkamp N. Asfour T. (2016). Unifying representations and large-scale whole-body motion databases for studying human motion. IEEE Trans. Robotics 32, 796–809. 10.1109/TRO.2016.2572685

[B45] Mathijssen G. Lefeber D. Vanderborght B. (2014). Variable recruitment of parallel elastic elements: series–parallel elastic actuators (SPEA) with dephased mutilated gears. IEEE/ASME Trans. Mechatr. 20, 594–602. 10.1109/TMECH.2014.2307122

[B46] Mende M. Scott M. L. van Doorn J. Grewal D. Shanks I. (2019). Service robots rising: how humanoid robots influence service experiences and elicit compensatory consumer responses. J. Market. Res. 56, 535–556. 10.1177/0022243718822827

[B47] Miller L. E. Zimmermann A. K. Herbert W. G. (2016). Clinical effectiveness and safety of powered exoskeleton-assisted walking in patients with spinal cord injury: systematic review with meta-analysis. Med. Devices (Auckland, NZ) 9:455. 27042146 10.2147/MDER.S103102 PMC4809334

[B48] Moreno J. C. Figueiredo J. Pons J. L. (2018). Exoskeletons for lower-limb rehabilitation, in Rehabilitation Robotics, eds Colombo R. Sanguineti V. (London; San Diego, CA; Cambridge, MA; Oxford: Elsevier), 89–99.

[B49] Moromizato K. Kimura R. Fukase H. Yamaguchi K. Ishida H. (2016). Whole-body patterns of the range of joint motion in young adults: masculine type and feminine type. J. Physiol. Anthropol. 35:23. 10.1186/s40101-016-0112-8 27716348 PMC5045662

[B50] Muraki Y. Ae M. Koyama H. Yokozawa T. (2008). Joint torque and power of the takeoff leg in the long jump. Int. J. Sport Health Sci. 6, 21–32. 10.5432/ijshs.6.21

[B51] Nelson A. C. Allen D. (1997). If you build them, commuters will use them: association between bicycle facilities and bicycle commuting. Trans. Res. Rec. 1578, 79–83. 10.3141/1578-10

[B52] Nelson G. Saunders A. Playter R. (2019). The petman and atlas robots at boston dynamics, in Humanoid Robotics: A Reference, eds Goswami A. Vadakkepat P. (Dordrecht: Springer), 169–186. 10.1007/978-94-007-6046-2_15

[B53] Paluska D. Herr H. (2006). The effect of series elasticity on actuator power and work output: implications for robotic and prosthetic joint design. Robot. Auton. Syst. 54, 667–673. 10.1016/j.robot.2006.02.013

[B54] Pieringer D. S. Grimmer M. Russold M. F. Riener R. (2017). Review of the actuators of active knee prostheses and their target design outputs for activities of daily living, in IEEE International Conference on Rehabilitation Robotics (ICORR) (London: IEEE), 1246–1253. 10.1109/ICORR.2017.8009420 28813992

[B55] Plooij M. Mathijssen G. Cherelle P. Lefeber D. Vanderborght B. (2015). Lock your robot: a review of locking devices in robotics. IEEE Robot. Automat. Mag. 22, 106–117. 10.1109/MRA.2014.2381368

[B56] Riener R. Rabuffetti M. Frigo C. (2002). Stair ascent and descent at different inclinations. Gait Post. 15, 32–44. 10.1016/S0966-6362(01)00162-X 11809579

[B57] Roaas A. Andersson G. B. (1982). Normal range of motion of the hip, knee and ankle joints in male subjects, 30–40 years of age. Acta Orthopaed. Scand. 53, 205–208. 10.3109/17453678208992202 7136564

[B58] Roebroeck M. Doorenbosch C. Harlaar J. Jacobs R. Lankhorst G. (1994). Biomechanics and muscular activity during sit-to-stand transfer. Clin. Biomech. 9, 235–244. 10.1016/0268-0033(94)90004-3 23916233

[B59] Schiller J. S. Kramarow E. A. Dey A. (2007). Fall injury episodes among noninstitutionalized older adults: United states, 2001-2003. Adv. Data 392, 1–16. 10.1037/e671852007-001 17953135

[B60] Schmidt K. Duarte J. E. Grimmer M. Sancho-Puchades A. Wei H. Easthope C. S.. (2017). The myosuit: Bi-articular anti-gravity exosuit that reduces hip extensor activity in sitting transfers. Front. Neurorobot. 11:57. 10.3389/fnbot.2017.00057 29163120 PMC5663860

[B61] Schurr S. A. Marshall A. N. Resch J. E. Saliba S. A. (2017). Two-dimensional video analysis is comparable to 3d motion capture in lower extremity movement assessment. Int. J. Sports Phys. Ther. 12:163. 28515970 PMC5380858

[B62] Seroussi R. E. Gitter A. Czerniecki J. M. Weaver K. (1996). Mechanical work adaptations of above-knee amputee ambulation. Arch. Phys. Med. Rehabil. 77, 1209–1214. 10.1016/S0003-9993(96)90151-3 8931539

[B63] Sinclair J. Hebron J. Hurst H. Taylor P. (2013). The influence of different cardan sequences on three-dimensional cycling kinematics. Hum. Movement 14, 334–339. 10.2478/humo-2013-0040

[B64] Sohn J. W. Kim G.-W. Choi S.-B. (2018). A state-of-the-art review on robots and medical devices using smart fluids and shape memory alloys. Appl. Sci. 8:1928 10.3390/app8101928

[B65] Spenko M. Buerger S. Iagnemma K. (2018). The DARPA Robotics Challenge Finals: Humanoid Robots To The Rescue, Vol. 121 Cham: Springer.

[B66] Stefanyshyn D. J. Nigg B. M. (1998). Contribution of the lower extremity joints to mechanical energy in running vertical jumps and running long jumps. J. Sports Sci. 16, 177–186. 10.1080/026404198366885 9531006

[B67] Sup F. Varol H. A. Mitchell J. Withrow T. J. Goldfarb M. (2009). Self-contained powered knee and ankle prosthesis: initial evaluation on a transfemoral amputee, in IEEE International Conference on Rehabilitation Robotics (ICORR) (Kyoto: IEEE), 638–644. 10.1109/ICORR.2009.5209625 PMC2836171 20228944

[B68] Tagliamonte N. L. Sergi F. Accoto D. Carpino G. Guglielmelli E. (2012). Double actuation architectures for rendering variable impedance in compliant robots: a review. Mechatronics 22, 1187–1203. 10.1016/j.mechatronics.2012.09.011

[B69] Torricelli D. Gonzalez J. Weckx M. Jiménez-Fabián R. Vanderborght B. Sartori M.. (2016). Human-like compliant locomotion: state of the art of robotic implementations. Bioinspirat. Biomimet. 11:051002. 10.1088/1748-3190/11/5/051002 27545108

[B70] Torricelli D. Gonzalez-Vargas J. Veneman J. F. Mombaur K. Tsagarakis N. del Ama A. J. (2015). Benchmarking bipedal locomotion: a unified scheme for humanoids, wearable robots, and humans. IEEE Robot. Automat. Mag. 22, 103–115. 10.1109/MRA.2015.2448278

[B71] Tudor-Locke C. E. Myers A. M. (2001). Methodological considerations for researchers and practitioners using pedometers to measure physical (ambulatory) activity. Res. Q. Exerc. Sport 72, 1–12. 10.1080/02701367.2001.10608926 11253314

[B72] Vanderborght B. Albu-Schäffer A. Bicchi A. Burdet E. Caldwell D. G. Carloni R. (2013). Variable impedance actuators: a review. Robot. Auton. Syst. 61, 1601–1614. 10.1016/j.robot.2013.06.009

[B73] Vaughan C. Davis B. O'Connor J. (1999). Dynamics of Human Gait. Cape Town: Kiboho Publishers.

[B74] Versluys R. Lenaerts G. Van Damme M. Jonkers I. Desomer A. Vanderborght B.. (2009). Successful preliminary walking experiments on a transtibial amputee fitted with a powered prosthesis. Prosthet. Orthot. Int. 33, 368–377. 10.3109/03093640902984587 19947821

[B75] Windrich M. Grimmer M. Christ O. Rinderknecht S. Beckerle P. (2016). Active lower limb prosthetics: a systematic review of design issues and solutions. Biomed. Eng. Online 15:140. 10.1186/s12938-016-0284-9 28105948 PMC5249019

[B76] Winter D. A. (1979). Biomechanics of Human Movement. New York, NY: John Wiley.

[B77] Winter D. A. (1980). Overall principle of lower limb support during stance phase of gait. J. Biomech. 13, 923–927. 10.1016/0021-9290(80)90162-1 7275999

[B78] Winter D. A. (2009). Biomechanics and Motor Control of Human Movement. Hoboken, NJ: John Wiley & Sons 10.1002/9780470549148

[B79] Winter D. A. Sidwall H. G. Hobson D. A. (1974). Measurement and reduction of noise in kinematics of locomotion. J. Biomech. 7, 157–159. 10.1016/0021-9290(74)90056-6 4837552

[B80] Wojtusch J. von Stryk O. (2015). Humod-a versatile and open database for the investigation, modeling and simulation of human motion dynamics on actuation level, in 2015 IEEE-RAS 15th International Conference on Humanoid Robots (Humanoids) (Seoul: IEEE), 74–79.

[B81] Woltring H. J. (1986). A fortran package for generalized, cross-validatory spline smoothing and differentiation. Adv. Eng. Softw. 8, 104–113. 10.1016/0141-1195(86)90098-7

[B82] Yan T. Cempini M. Oddo C. M. Vitiello N. (2015). Review of assistive strategies in powered lower-limb orthoses and exoskeletons. Robot. Auton. Syst. 64, 120–136. 10.1016/j.robot.2014.09.032

[B83] Yang T. Westervelt E. R. Schmiedeler J. P. Bockbrader R. A. (2008). Design and control of a planar bipedal robot ernie with parallel knee compliance. Auton. Robots 25:317 10.1007/s10514-008-9096-5

[B84] Yang X. She H. Lu H. Fukuda T. Shen Y. (2017). State of the art: bipedal robots for lower limb rehabilitation. Appl. Sci. 7:1182 10.3390/app7111182

[B85] Yeadon M. R. King M. A. Wilson C. (2006). Modelling the maximum voluntary joint torque/angular velocity relationship in human movement. J. Biomech. 39, 476–482. 10.1016/j.jbiomech.2004.12.012 16389087

[B86] Zavorsky G. S. Tomko K. A. Smoliga J. M. (2017). Declines in marathon performance: sex differences in elite and recreational athletes. PLoS ONE 12:e0172121. 10.1371/journal.pone.0172121 28187185 PMC5302805

[B87] Zhong Y. Fu W. Wei S. Li Q. Liu Y. (2017). Joint torque and mechanical power of lower extremity and its relevance to hamstring strain during sprint running. J. Healthcare Eng. 2017:7. 10.1155/2017/8927415 29065661 PMC5529660

